# Design and Speed-Adaptive Control of a Powered Geared Five-Bar Prosthetic Knee Using BP Neural Network Gait Recognition

**DOI:** 10.3390/s19214662

**Published:** 2019-10-27

**Authors:** Yuanxi Sun, Rui Huang, Jia Zheng, Dianbiao Dong, Xiaohong Chen, Long Bai, Wenjie Ge

**Affiliations:** 1 State Key Laboratory of Mechanical Transmission, Chongqing University, Chongqing 400044, China; rui.huang@cqu.edu.cn (R.H.); chenxh@cqu.edu.cn (X.C.); bailong@cqu.edu.cn (L.B.); 2 School of Advanced Manufacturing Engineering, Chongqing University of Posts and Telecommunications, Chongqing 400065, China; zhengjia@cqupt.edu.cn; 3 School of Mechanical Engineering, Northwestern Polytechnical University, Xi’an 710072, China; dongdianbiao@mail.nwpu.edu.cn (D.D.); gwj@nwpu.edu.cn (W.G.); 4 Department of Mechanical Engineering, Vrije Universiteit Brussel, 1050 Brussels, Belgium

**Keywords:** prosthetic control, neural network, prosthetic knee, geared five-bar mechanism

## Abstract

To improve the multi-speed adaptability of the powered prosthetic knee, this paper presented a speed-adaptive neural network control based on a powered geared five-bar (GFB) prosthetic knee. The GFB prosthetic knee is actuated via a cylindrical cam-based nonlinear series elastic actuator that can provide the desired actuation for level-ground walking, and its attitude measurement is realized by two inertial sensors and one load cell on the prosthetic knee. To improve the performance of the control system, the motor control and the attitude measurement of the GFB prosthetic knee are run in parallel. The BP neural network uses input data from only the GFB prosthetic knee, and is trained by natural and artificially modified various gait patterns of different able-bodied subjects. To realize the speed-adaptive control, the prosthetic knee speed and gait cycle percentage are identified by the Gaussian mixture model-based gait classifier. Specific knee motion control instructions are generated by matching the neural network predicted gait percentage with the ideal walking gait. Habitual and variable speed level-ground walking experiments are conducted via an able-bodied subject, and the experimental results show that the neural network control system can handle both self-selected walking and variable speed walking with high adaptability.

## 1. Introduction

The prosthetic knee is a vital apparatus for the rehabilitation of lower limb amputations, which can enable above-knee amputees to regain walkability in daily life. Compared with purely passive prosthetic knee joints, the powered prosthetic knee joints can provide the amputees with active actuation when needed, thus improving the walking gaits of amputees. To achieve satisfactory performance, the mechanical structure, actuating device, and control system of the powered prosthetic knee must be designed appropriately according to their respective requirements and mutual relations.

The control system, which works as the “brain” of the powered prosthetic knee, directly determines the response of the prosthetic knee. Therefore, various control methods, such as the adaptive control [1,2,3,4], finite state control [5,6], expect system control [7], central pattern generator based control [8], EMG based control [9,10], neural network control [11,12,13], etc., have been proposed to implement and further improve the control performance of the prosthetic knee. Compared with other parametric model-based control methods, the neural network is a data-driven system, which has advantages of fewer difficulties in more accurate system modeling and stronger capabilities of handling known abnormal inputs when adequately trained. E. Tileylioğlu et al. proved by experiments that the neural network-based approach can generate slightly better results than conventional rule-based algorithms [14].

Generally, there are two ways to implement the neural network control of the prosthetic knee:

(1) Use the neural network to learn the underlying relations between inputs and control parameters of the actuating device, thus controlling the prosthetic knee [15];
(2) Use the neural network to predict motions of the amputated leg, based on which the specific control instructions of the actuating device is generated [16].

Compared with the former efficient application-specific method, the latter one is more versatile.

Many different sources of inputs can be used for the neural network control of prosthetic joints. For example, the surface electromyography (EMG) is an electrical activity generated by the muscle cell, which can be detected via surface electrodes and used for limb pattern recognition. S. Li et al. used EMG signals from five muscles of the lower limb as inputs of the trained neural network, which can successfully identify three different level walking speeds with a general recognition rate of 90.48% [17]. L. Liu et al. used the extracted features of the EMG signals from able-bodied subjects via the principal component analysis as inputs of the neural network, thereby successfully recognizing locomotion patterns such as walking up/down hills, walking up/down stairs, etc. [18]. Similar to Liu [18], R. Oweis used features extracted by sample entropy, fourth-order cepstral coefficient, root mean square and waveform length, as inputs of the neural network, thus realizing five human forearm hand gestures recognition with an average accuracy of 96.7% [19].

In addition to EMG signals, signals of mechanical sensors such as accelerometers, gyroscopes, load cells, etc., are also widely used in neural network control of the prosthetic joint. Compared with sEMG-based control, sensor-based control has the advantage of convenience in use, lower cost, and better reliability [20,21]. For example, M. Islam et al. used velocity and segmentation angle of the ankle as inputs of the neural network, which can automatically detect the gait mode of the powered ankle-foot orthosis [22]. K. Ekkachai et al. proposed a neural network predictive control of the MR damper prosthetic knee with inputs of the knee angle and control voltage, which can provide amputees with better knee angle trajectories than conventional open-loop controllers [23]. Apart from using only kinematic data, dynamics data such as joint forces can also be used for the neural network control. U. Demir et al. used the neural network with inputs of the angle and force of the Physiotherabot to estimate impedance parameters during manual therapy, which can make the Physiotherabot suitable for the personal robotic treatment of different patients [24]. J. Jung et al. used the ground reaction force and joint angles of the sound leg as inputs of the neural network, which can recognize current phase gait and generate predicted motions of the unsound leg [25]. Moreover, G. Li developed a novel wearable sensor shoe, which can measure force situations of the foot, and used 3D force situations of the foot as inputs of the neural network to predict motions of the measured limb. Unlike the conventional limb sensing system, this sensing shoe is more convenient [26].

There is also some indirect information that can be used for motion recognition of lower limbs. For example, D. Joshi et al. predicted the motion of the residual limb via the cross-correlation coefficient obtained from sixteen healthy adult males [27], while H. Vallery et al. used statistical regression [28] between the sound leg and the residual limb to control the prosthetic knee. In addition, P. Kutilek used the angle-angle diagram between hip, knee, and ankle joints as inputs for the neural network to predict limb motions [29].

In this paper, the authors intend to use the neural network to develop a speed-adaptive control of a previously proposed GFB prosthetic knee concept [30]. However, by thoroughly analyzing the above literature, three potential shortcomings of current neural network control methods for the powered lower-limb prosthesis could be found as follows:

(1) Current neural network control methods did not take into account the past walking gait, which contains the time information that has been proven essential to the improvement of the overall control performance [31,32] and the compensation for inherent system noise [22].
(2) Current neural network control methods directly predicted the prosthetic locomotion, which will make it hard to modify the gait output for the adaptability of different walking conditions without retraining the neural network.
(3) Current neural network control methods often used data from the sound limb of the amputee as inputs, which will lose versatility in dealing with asymmetrical walking gaits such as acceleration or deceleration.

To solve these shortcomings, this paper tries to use the current walking gait as well as the past walking gait of only the prosthetic knee as inputs of the neural network, and then matches the predicted walking gait percentage with the ideal gait database that contains walking gaits corresponding to different walking speeds to generate specific knee motions for the motor control, thereby improving the speed-adaptive and asymmetrical walking effects of the prosthetic knee as well as the modifiability of the neural network control.

The rest of this paper is organized as follows: Section 2 presented the structure and the actuation design of the GFB prosthetic knee, and Section 3 is the speed-adaptive neural network control of the GFB prosthetic knee. Section 4 shows the experimental verification of the control algorithm, and Section 5 concludes the paper.

## 2. Design of the Powered GFB Prosthetic Knee 

### 2.1. Structure Design

**Figure 1:**
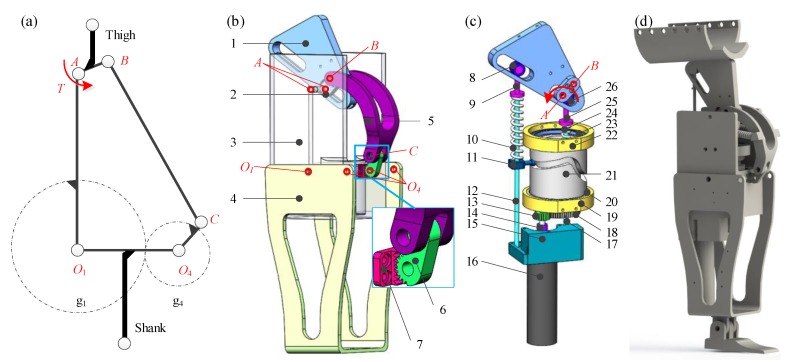
(**a**) Schematic diagram, (**b**) structure, (**c**) actuation mechanism, and (**d**) CAD model of the GFB prosthetic knee. 1-level arm (fixed on linkage AB), 2-linkage AB (thigh linkage), 3-linkage $O_{1}A$ (frame), 4-linkage $O_{1}O_{4}$ (shank linkage), 5-linkage BC, 6-linkage $CO_{4}$ (incomplete gear $g_{4}$ centered at hinge $O_{4}$), 7-incomplete gear $g_{1}$ (fixed on linkage $O_{1}A$), 8-bearing, 9-spring fastening, 10-helical spring, 11-cam roller, 12-guide, 13-transmission gear, 14-coupling, 15-motor cabinet (fixed on frame), 16-motor, 17-guide, 18-transmission gear, 19-bearing chock, 20-bearing, 21-cylindrical conjugate cams (two cam grooves on outer/inner walls of the cylinder), 22-bearing chock, 23-bearing, 24-helical spring, 25-spring fastening, 26-bearing.

The GFB prosthetic knee consists of a GFB mechanism and a cam-based nonlinear series elastic actuator. Specific theoretical modeling and formula derivation are presented in [30,33]. Its schematic diagram, structure, actuation mechanism, and the CAD model are all depicted in Figure 1.

The dimension of the GFB mechanism is optimized by the improved genetic annealing algorithm [34] to implement the bionic human knee centrode. The linkage $O_{1}A$ works as the frame to enable the installation of the actuating component in its cavity. The linkage AB acts as the thigh bar and the linkage $O_{1}O_{4}$ is the shank bar. The linkage BC is modified as curved to avoid its motion interference [35] with the actuating component. The endpoint $O_{4}$ of the linkage $O_{4}C$ is designed as an incomplete gear, which can mesh with the incomplete gear centered at $O_{1}$ on the linkage $O_{1}A$ to form the gear mechanism.

The actuating component of the GFB prosthetic knee is a cam-based nonlinear series elastic actuator, whose dimensional parameters are optimized to enable the prosthetic knee to realize natural level-ground walking gait while the energy consumption of the actuator is minimized. The motor drives the conjugate cylindrical cams to rotate via the gear transmission (5:1), which can deform two helical springs that reciprocate along the frame slides to actuate the thigh linkage AB during walking.

The specifications of the powered GFB prosthetic knee are listed in Table 1.

### 2.2. Motor Control

**Figure 2:**
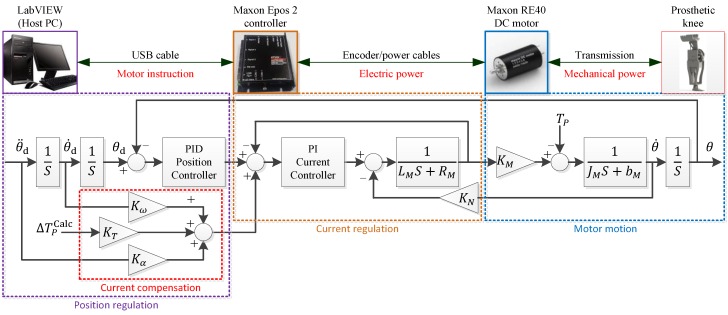
Motor control system (feedforward current compensations are provided to deal with inertial-related, speed-related and load-related changes in the prosthetic system). $L_{m}$ and $R_{m}$ are the inductor and the resistor of the motor, and $J_{m}$ and $b_{m}$ are the moment of inertial and damping of the motor and transmissions; $K_{M}$ and $K_{N}$ are the torque constant and the speed constant of the motor, respectively; $K_{\omega }$, $K_{\tau }$ and $K_{\alpha }$ are corresponding gains of feedforward current compensations; $T_{p}$ is the needed actuating torque of the prosthetic knee, and $\Delta T_{P}^{Calc}$ is the calculated prosthetic torque changes by real-time inverse dynamics of the prosthetic knee.

The motor system of the GFB prosthetic knee is shown in Figure 2.

The hardware of the motor control system consists of a host PC (LabVIEW), a motor controller (Epos 2 50/5, Maxon, Sachseln, Switzerland), and a brushed DC motor (RE 40, 24V, Maxon, Sachseln, Switzerland) with an integrated reducer (GP 42, 26:1, Maxon, Sachseln, Switzerland). The host PC communicates with the Maxon Epos 2 controller via a USB cable based on the LabVIEW-Maxon EPOS instrument Driver. The DC motor is connected to the Maxon Epos 2 controller via a 3-channel differential incremental encoder (ML, 512-line, Maxon, Sachseln, Switzerland) as well as a power cable. 

The motor control architecture is finalized by the cascade of a PI (Proportional-Integral) current regulation and a PID (Proportional-Integral-Derivative) position regulation. The sampling frequencies of the PI current regulation and the PID position regulation are 1 kHz and 500 Hz, respectively. Because the prosthetic knee angle is mechanically related to the output angle of the cam-based nonlinear series elastic actuator, the input of the prosthetic control is set as the desired output angle $\theta_{d}$, angular velocity ${\dot{\theta }}_{d}$, and angular acceleration ${\ddot{\theta }}_{d}$ of the cam-based nonlinear series elastic actuator, and the output of the control is the actual output angle θ of the cam-based nonlinear series elastic actuator. Additional feedforward compensations are provided for the current regulation of the motor to provide necessary actuating torque for the prosthetic knee in addition to the primary actuation of the motor system. The acceleration feedforward and the speed feedforward are added to deal with the moment of inertial related changes and the speed-related damping in the motor system, while the torque feedforward is meant for the compensation of changes in prosthetic load.

### 2.3. Attitude Measurement

**Figure 3:**
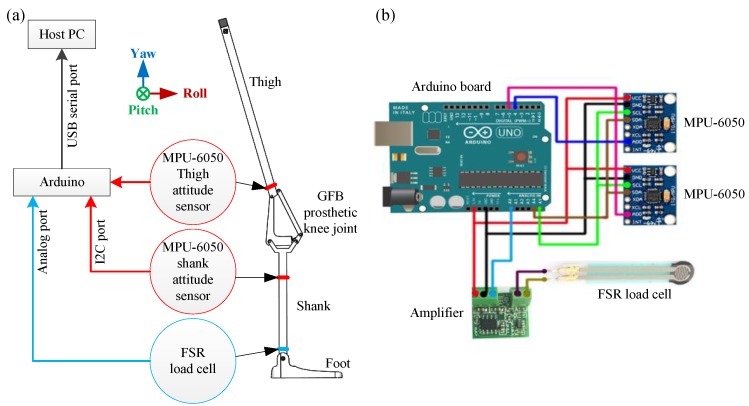
Attitude measurement platform: (**a**) schematic diagram; (**b**) hardware diagram.

The attitude measurement of the GFB prosthetic knee is to use mechanical sensors to obtain the locomotion data for the neural network control. The specific platform is shown in Figure 3, which consists of an Arduino Uno board, dual IMU sensors (MPU-6050, InvenSense, San Jose, CA, USA) and one FSR load cell (Force Sensing Resistor, Interlink Electronics, Camarillo, CA, USA). The Arduino UNO development board works as a sensor hub to communicate with the host computer. The FSR load cell is installed at the bottom of the prosthetic knee to distinguish the stance phase and the swing phase in the level-ground walking cycle. Dual MPU-6050 sensors, which are individually accessed via the I2C address 0x68 (the one whose AD0 port is set high) and 0x69 (the one whose AD0 port is configured low), are installed on the upper and lower linkages of the GFB prosthetic knee to measure the motions of the lower extremity. The Yaw, Pitch, and Roll data of the thigh and the shank can be directly obtained by the DMP (Digital Motion Processor) within two MPU-6050 sensors, the sampling frequency of which are set as 100 Hz.

**Figure 4:**
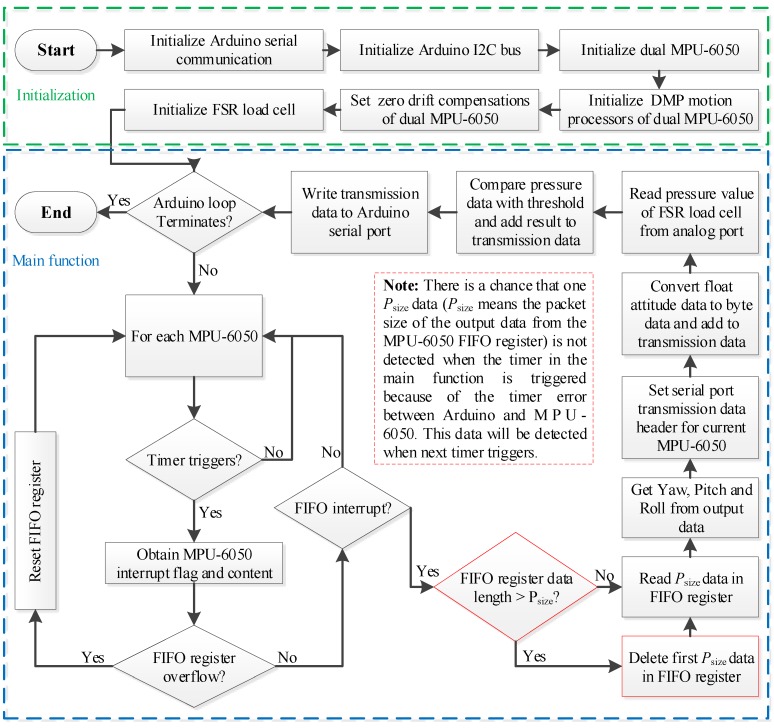
Attitude measurement of the powered geared five-bar (GFB) prosthetic knee.

The overall attitude measurement process of the GFB prosthetic knee is shown in Figure 4. To identify which sensor the transmitted data belongs to, the Header Byte of the transmitted data from the thigh MPU-6050 and the shank MPU-6050 are set to 0x69 and 0x0c, respectively. The current walking phase of the prosthetic knee can be obtained by identifying the walking phase Flag. When in the stance phase, the value of the walking phase Flag is 1; when in the swing phase, the Flag value is 0. The details of the transmitted data are listed in Table 2.

### 2.4. Parallel Implementation of the Attitude Data Processing and the Motor Control

**Figure 5:**
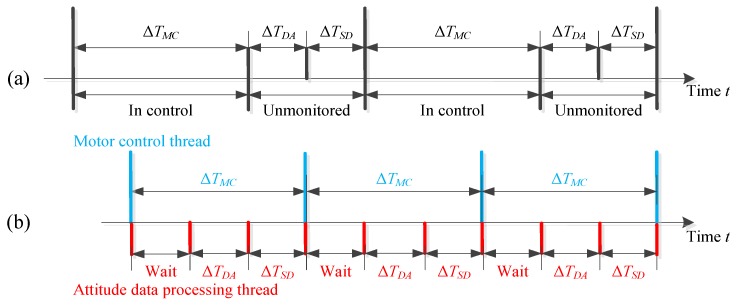
(**a**) Serial and (**b**) parallel implementation of the attitude data acquisition, processing, and motor control. $\Delta T_{MC}$ represents the period the motor control command is sent and executed; $\Delta T_{SD}$ represents the period to collect the transmitted attitude data from Arduino; $\Delta T_{DA}$ is the period required for the host PC to process the transmitted data.

If the motor control function and the attitude data acquisition/processing function are operated in the serial mode, the motor will be unmonitored at some specific period. The attitude data acquisition/processing period must be restricted below millisecond levels to ensure control timeliness, which will increase the design difficulty of both the software and hardware systems, as shown in Figure 5a. However, if the attitude data acquisition/processing function and the motor control function are run in parallel as shown in Figure 5b, the motor will remain monitored during the whole walking cycle. The only issue that must be seriously taken care of in the parallel operation is to ensure the data synchronization between threads, which can be practically implemented by local variables (thread data sharing) and semaphores (thread data protection). In this paper, the control frequency of the prosthetic controller is set as 50 Hz.

## 3. Speed-Adaptive Neural Network Control of the Powered GFB Prosthetic Knee

### 3.1. Gait Analysis and Feature Extraction

Different people have different walking gaits, which are strongly related to their own heights, weights, walking speeds, and walking habits, etc. To study the proper gaits of different people, gait data of ten able-bodied subjects with different physical characteristics distributions (listed in Table 3) was collected for analysis (written consent forms were obtained prior to data collection). 

**Figure 6:**
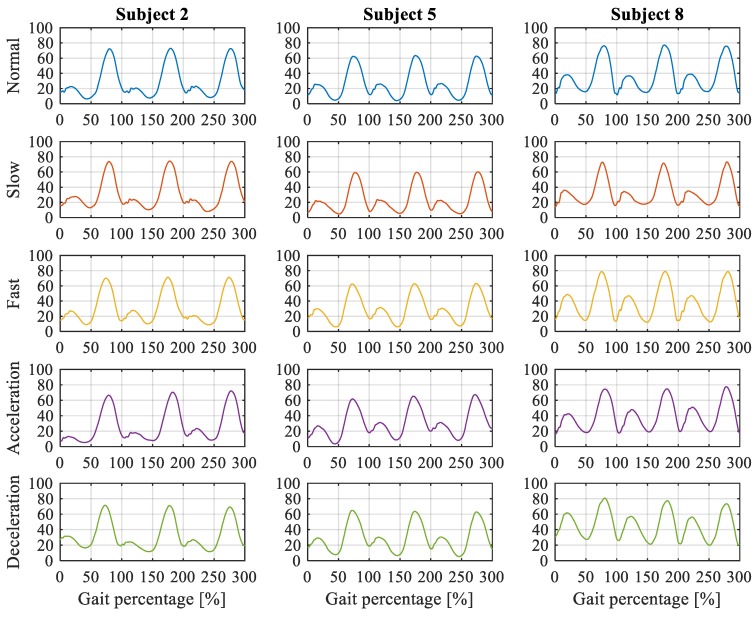
Knee angles of subject 2, 5 and 8 in different walking conditions.

Figure 6 shows the continuous gait cycles of the representative subject 2, 5 and 8 under normal, fast, slow, accelerated and decelerated walking situations. The speed of the normal walking is 1.3 m/s, while the fast and the slow walking speeds are 50% and 150% of the normal speed, respectively. The acceleration and deceleration processes refer to the recorded process from the slow walking to the fast walking, and from the fast walking to the slow walking, respectively. 

It can be noticed from Figure 6, that different subjects have different walking gaits, such as the peak stance/swing angles during a walking cycle, the lowest knee angles during a walking cycle, the stance/swing cycle percentages during a walking cycle, the knee angle standard deviation during a walking cycle, etc. For example, the peak knee angle of the subject 5 is smaller than that of subjects 2 and 8 in every walking conditions, while the stance peak knee angle of the subject 8 is much larger than that of subjects 2 and 5 in every waking condition. In addition to the gait differences between individuals, the gait of the subject itself at different walking speeds also varies. For instance, the stance phase of the subject 2 in fast walking is a little longer than that in slow walking.

Therefore, to improve the versatility and practicality of the powered GFB prosthetic knee joint, this paper uses the recorded gait data of all the able-bodied subjects in Table 3 as the reference gaits and store them in the ideal gait database for subsequent knee control. These subjects are of balanced age, height, weight, and gender distribution, which can bring in expected gait control effect.

However, variable walking conditions for different people such as different walking speeds or acceleration/deceleration show certain differences, which must be evaluated and identified accurately for the following neural network control. Compared with the time-domain characteristics, the statistical characteristics of gait signals can be used to perform fast extraction of gait movement laws and motion trends in embedded development. Specific statistical features of gait signals are listed in Table 4.

The Mean, the Median, and the Mode mathematically mean the average of a sequence of inputs, which can be used to implement the noise reduction for the original input. As the Mean has a better effect in smoothing the original input than the other two methods, this paper used the Mean (MN) as one of the key inputs for the gait feature extraction. 

The Total Distance, the Variance, the Standard Deviation, and the Coefficient of Variation can be used to describe the degree of divergence of the input signal. The difference between these four methods is that they are sensitive to the magnitude of the signal fluctuation amplitude while the identifications of the signal trend stay completely consistent. Therefore, this paper used the Standard Deviation (SD) for the gait feature extraction due to its moderate sensitivity in systematic testing.

The Skewness is a measure of input signal asymmetry while the Kurtosis measures whether the input signal distribution is sharper or flatter than the normal distribution. As the Standard Deviation is able to reflect the magnitude of the input, this paper used the Skewness (SKE) for the subsequent gait feature extraction.

The Slope Count (SC) and the Slope Zero Crossing (SZC) can be used to measure the trend change of the input signal, which are essential to the identification of variable speed walking such as acceleration and deceleration. Therefore, these two parameters are all used in the subsequent gait feature extraction.

**Figure 7:**
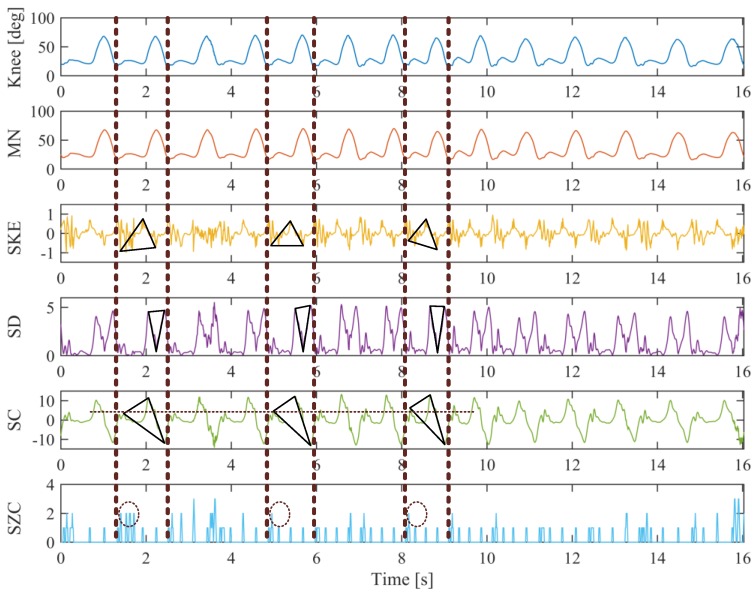
Feature extraction example of the “acceleration->fast walking->deceleration” walking of subject 1.

Figure 7 shows the feature extraction of the “acceleration->fast walking->deceleration” walking of the subject 1 by using the five criteria discussed above. The knee gait input consists of 14 cycles that last about 16 s. It can be directly noticed from the knee angle curve that the duration of one cycle at different walking speeds differs. The duration of one cycle in the fast walking is shorter than that in the slow walking, i.e., this information can be used for the identification of different walking speeds. However, the acceleration or deceleration phase cannot be easily identified by the original knee angle curve, which must be implemented with the help of other feature curves. For instance, the acceleration or constant speed or deceleration can be efficiently recognized via the SKE curve. In the acceleration stage, the slope of the triangle formed by the start lowest point, the middle highest point and the end lowest point is much higher than that in the constant speed walking, while it is much lower in the deceleration stage. These same characteristics can also be revealed from the SD, SC and the SZC curves. Therefore, by using these curves for gait stage and transition identification, the actual walking status of the patients can be obtained to realize the adaptive control of the GFB prosthetic knee.

### 3.2. Following Control Strategy

**Figure 8:**
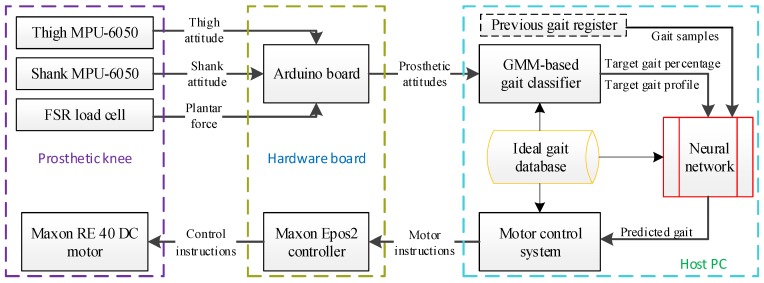
Following control strategy of the powered GFB prosthetic knee.

In order for the prosthetic knee to follow the thigh motion of the amputee, this paper proposed a novel neural-network-based gait database matching method to achieve the specific control, as shown in Figure 8.

**Figure 9:**
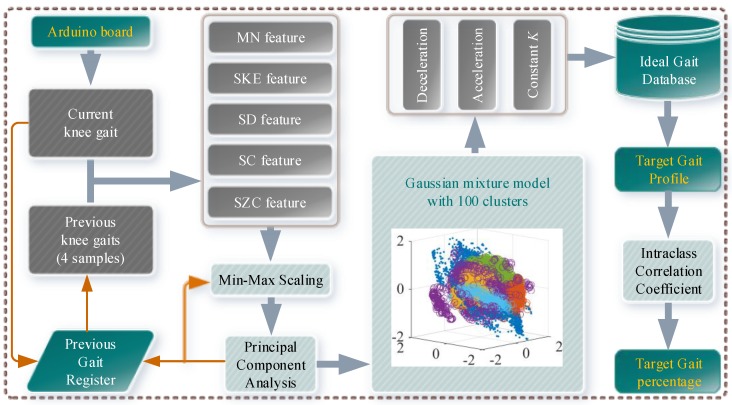
Gaussian mixture model-based gait classifier.

The real-time attitude of the prosthetic knee is transmitted to the host PC via the Arduino board, and the current speed ratio and the gait percentage of the prosthetic knee are calculated by the Gaussian mixture model-based gait classifier, as shown in Figure 9. 

The workflow of the Gaussian mixture model-based gait classifier (GMMGC) is as follows: the GMMGC first obtains the current knee angle and the last four samples of previous knee angles from the Arduino board and the Previous Gait Register (PGR), respectively. Then the Mean (MN), the Skewness (SKE), the Slope Count (SC), and the Slope Zero Crossing (SZC) features of the total five input samples are extracted. Because the amplitude of different feature differs (for example, the MN feature is an order of magnitude larger than others), each feature is normalized via the Min-Max Scaling method by extracting their previous four samples from the PGR to ensure that each feature will have the same impact on the final classification result. In order to improve the efficiency of the gait classification, this paper used the Principal Component Analysis (PCA) to perform the dimension reduction of input knee gait features. To ensure all the variability of the dimensionally reduced features is larger than 90%, the first three components are adopted for the subsequent gait classification.

To perform reliable and fast classification of input gaits, this paper compared some typical multi-label classification methods (such as the Discriminant Analysis, the Support Vector Machines, the K-Nearest Neighbor Classifiers, and the Naive Bayes Classifiers, the Stochastic Gradient Descent classifiers, etc.) and some typical unsupervised classification methods (such as the Expectation-Maximization, the K-means, the Gaussian mixture model, etc.) via a training data (121903 sets, 100 labels/clusters of 10 subjects with 10 walking gaits) in MATLAB. The results show that the Gaussian kernel Support Vector Machine (hyperparameters are optimized via the Bayesian optimization as [Coding: One VS. One, BoxConstraint: 895.69, Kernel Scale: 0.68021, Standardize: true]) achieved >95% classification accuracy, while the K-Nearest Neighbor achieved >99.99% classification accuracy. However, the K-Nearest Neighbor showed a low posterior probability. As for the unsupervised classification methods, the Gaussian mixture model achieved the highest >85% classification accuracy with a high posterior probability. Because the Gaussian kernel Support Vector Machine used the One VS. One coding, there will be 100×99/2=4950 binary Support Vector Machine learners, which is not beneficial for the subsequent embedded integration into the development board. Although the Naive Bayes Classifier is simple enough for subsequent embedded integration, it could provide only >68% classification accuracy. Therefore, this paper finally adopted the Gaussian mixture model for fast gait classification. The classification result of the training data (feature dimensions reduced from 5 to 3 by PCA) via the trained Gaussian mixture model is depicted within Figure 9.

After the classification of the input knee gait, the target gait profile can be obtained by matching the classification result with the Ideal Gait Database (IGD). The ideal gait profiles corresponding to different walking speeds in the IGD are normalized for adaptability. Then the gait percentage can be quickly calculated by using the intraclass correlation coefficient (ICC), i.e., compare a small time window of the walked gait with the target gait profile to find the gait cycle percentage, as shown in Equation (1):

(1) $$\left[t\right]=\underset{1\leq t\leq N-5}{\min}\frac{\sum_{i=1}^{n}\left(\theta_{i-n}-m\right)\left(G_{t+i-n+1}-m\right)}{\left(n-1\right)S}$$

where

$$m=\frac{\sum_{1}^{n}\left(\theta_{i-n}+G_{t+i-n+1}\right)}{2n},S=\sqrt{\frac{\sum_{i=1}^{n}{\left(\theta_{i-n}-m\right)}^{2}+\sum_{i=1}^{n}{\left(G_{t+i-n+1}-m\right)}^{2}}{2n-1}}$$

In Equation (1), t is the gait cycle percentage; $G_{t}$ represents the data at the t% gait cycle percentage of the kth ideal gait profile; $\left[\theta_{-n},\theta_{-n+1},\ldots ,\theta_{-1},\theta_{0}\right]$ are the past n−1 collected discrete actual prosthetic knee angles and the current prosthetic knee angle.

Then by using the calculated gait percentages and previous four percentages in the target gait profile as inputs (because the sampling frequency of the prosthetic controller is 50 Hz, the total locomotion period of these five attitudes will be 100 ms, which is sufficient for pattern recognition), the neural network will output the predicted gait percentage of the prosthetic knee. Finally, by matching the predicted gait percentage with the target gait profile, the predicted prosthetic knee posture is generated. The host PC will then send corresponding motor instructions to the motor system to actuate the prosthetic knee.

**Figure 10:**
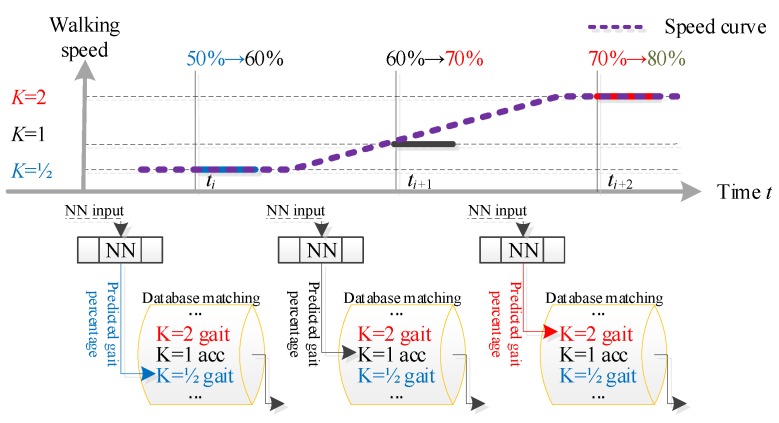
Typical operating state during control.

For example, in a theoretical control state as shown in Figure 10, speed ratios and gait percentages calculated by Equation (1) are [K=1/2,  50%] at $t_{i}$, [K=1,  60%] at $t_{i+1}$, and [K=2, 70%] at $t_{i+2}$, respectively. At $t_{i}$, the neural network predicted gait percentage is 60%. Then the next prosthetic knee attitude is obtained by matching 60% gait percentage with the K=1/2 ideal gait data; at $t_{i+1},$ the next prosthetic knee attitude is generated by matching the neural network predicted 70% gait percentage with the K=1 acceleration ideal gait; and so on.

To avoid abnormity in practical conditions, measured K is pre-processed by limiting it to values of only 1/4, 1/2, 3/4,1, 5/4, 3/2, 7/4, and 2 via approximation. When the measured K is greater than 2, it is limited to 2 due to level-grounding walking limitation. When the measured K is less than 1/4 but higher than the given threshold $T^{*}$, it remains as 1/4 to continue the prosthetic knee locomotion. When the measured K is below the given threshold $T^{*}$, the pre-processing procedure calls the locomotion initiation/termination functions for further investigation. In this paper, the threshold $T^{*}$ is obtained via practical experiments.

The prosthetic knee initiation: when the device is turned on, it will automatically home to the state of zero knee flexion. When the flexion angle of the prosthetic knee reaches zero, the prosthetic knee will stand by until the measured speed ratio K is greater than the initiation threshold ($T_{I}{}^{*}=0.18$). 

The prosthetic knee termination: when the measured speed ratio is lower than the termination threshold ($T_{T}{}^{*}=0.09$), the prosthetic knee will automatically home to the state of zero knee flexion, and the motor shaft will be locked by the motor controller to enable the stance support of the prosthetic knee.

### 3.3. Neural Network Design

**Figure 11:**
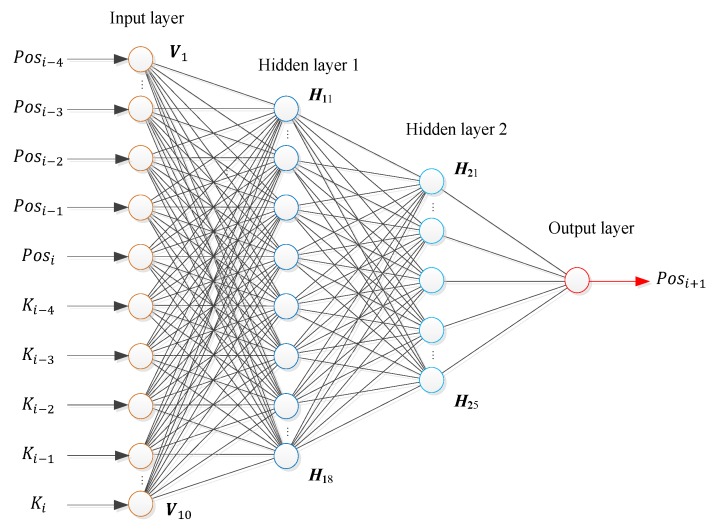
Structure of the BP neural network.

In this paper, a BP neural network is utilized for the neural network control, as shown in Figure 11. 

The input layer of the BP neural network has 10 nodes that correspond to 10 input variables, namely, last four and current gait percentages Pos and the speed ratios K of the prosthetic knee (5 points in 100 ms are used as the input variable of the neural network because a 100 ms time window contains enough data information for pattern recognition, and the amount of data processed is not particularly large. Using 7, 9, or more points as the inputs will not improve the detection accuracy). Since the control system cannot be expressed in the form of a continuous function, the BP neural network is set as the double hidden layer 8 × 5 (Compared with 10 × 5 and 8 × 3 structures and other configurations, the 8 × 5 hidden layer structure has the best correlation between the predicted value and the expected value after testing). The activation function of the neural network is the S-type function, and the output layer is a single node (the predicted Pos value).

### 3.4. Training and Performance Test of the Neural Network

**Figure 12:**
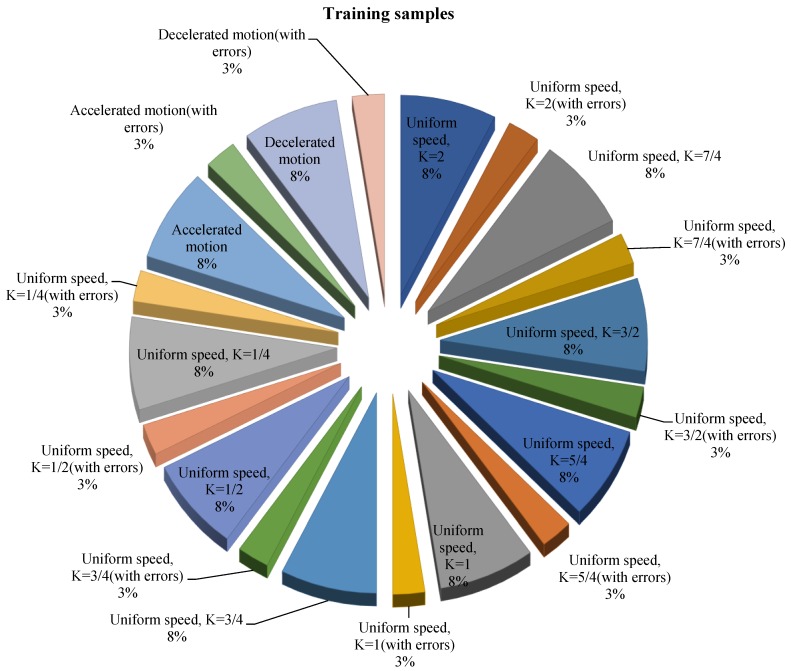
The distribution of training data for the neural network.

To ensure that the neural network has excellent generalization performance and a high recognition rate, 3500 training samples of ten different categories are used in this paper. The training data in each category contains 87.5% correct data and 12.5% error data (inputs are modified with slight noise and accidental errors), as shown in Figure 12. Specific training data is collected via recorded gait from Section 3.1. The reference speed (i.e., K=1) is 1.3 m/s.

**Figure 13:**
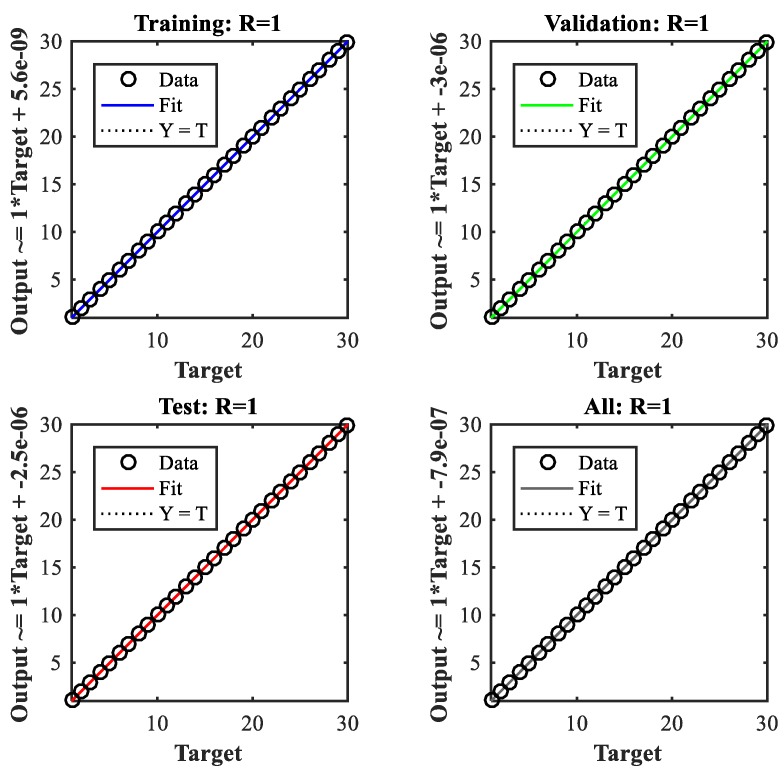
Regression of the training, validation, and test of the neural network.

By using 500 separate sets of validation data and another 500 different sets of test data, the regression of training, validation, and test are shown in Figure 13. The overall performance of the trained neural network reaches 1.19 × 10^−10^, which proves that the designed neural network has the right prediction and correction over the training data.

### 3.5. Overall Control of the GFB Prosthetic Knee

**Figure 14:**
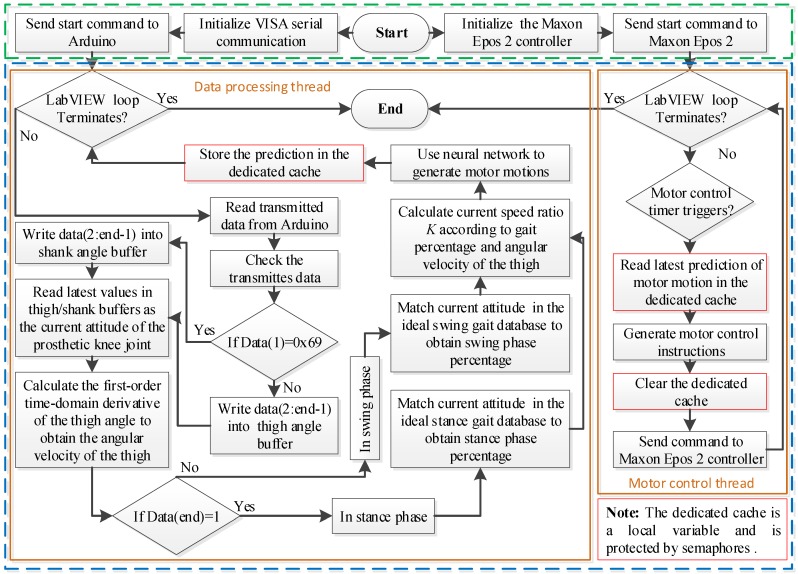
Overall control of the powered GFB prosthetic knee.

The overall control flowchart of the GFB prosthetic knee is shown in Figure 14. The attitude data acquisition/processing thread and the motor control thread are run in parallel, while their thread data is synchronized by local variables.

## 4. Reliability Analysis and Experimental Evaluation

### 4.1. Comparison with Typical Gait Prediction Methods

To study the prediction effect of the proposed BP neural network-based gait detection method, this paper compared it with typical gait detection methods: Adaptive Network-based Fuzzy Inference System (ANFIS), Gaussian process regression (GPR), and Support Vector Machine (SVM). The parameters of ANFIS for comparison are as follows: Numbers of clusters: 5; Radii of clusters: 0.5; Range of influence of the cluster center: 0.5; Squash factor: 1.25; Acceptance ratio: 0.5; Rejection ratio: 0.15. The parameters of GPR for comparison are as follows: Explicit basis in the GPR model: constant; Kernel function: squared exponential kernel. The parameters of SVM for comparison are as follows: Kernel function: linear; Kernel scale: 1.

**Figure 15:**
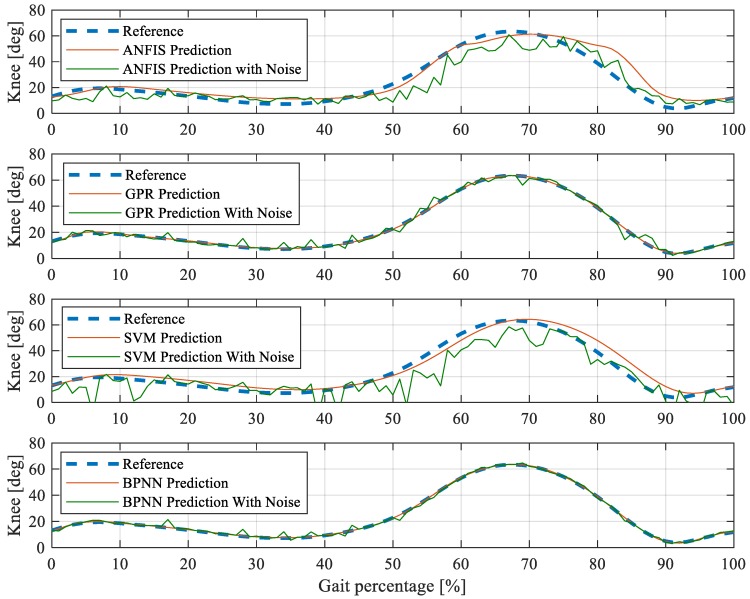
BP neural network gait detection comparison with adaptive network-based fuzzy inference system (ANFIS), gaussian process regression (GPR), and support vector machine (SVM).

The BP neural network, the ANFIS, the GPR, and the SVM are all trained with the same training dataset. To evaluate the prediction ability of these methods, a test reference gait and its noised one are utilized to check the prediction accuracy. The results are depicted in Figure 15. It can be known from Figure 15 that the prediction accuracies of ANFIS for both the reference gait and the noised one are not satisfactory. The root means square errors (RMSEs) of the ANFIS predicted gaits to the reference gait and the noised reference gait are 5.4179 deg and 6.9074 deg, respectively. The prediction of SVM for the reference gait is close to expected, but the prediction of the noised one is poor. The RMSEs of the SVM predicted gaits to the reference gait and the noised reference gait are 2.9665 deg and 12.3637 deg, respectively. This result indicates that the SVM method is susceptible to noise, which limits its practical effects if no additional robust algorithms are provided.

It can be noticed from Figure 15 that the GPR has good prediction accuracies for both the reference gait and the noised one. The RMSEs of the GPR predicted gaits to the reference gait and the noised reference gait are 0.3518 deg and 2.0857 deg, respectively. However, compared with the GPR, the BP neural network performs better in predicting both the reference gait and the noised one. The RMSEs of the BP neural network predicted gaits to the reference gait and the noised reference gait are 0.2165 deg and 2.4831 deg, respectively. In addition, compared with GPR, the BP neural network consumes much fewer computing resources in embedded hardware systems. The use of BP neural network in embedded systems can effectively improve the gait detection accuracy without occupying additional computing performance.

### 4.2. Reliability Analysis

**Figure 16:**
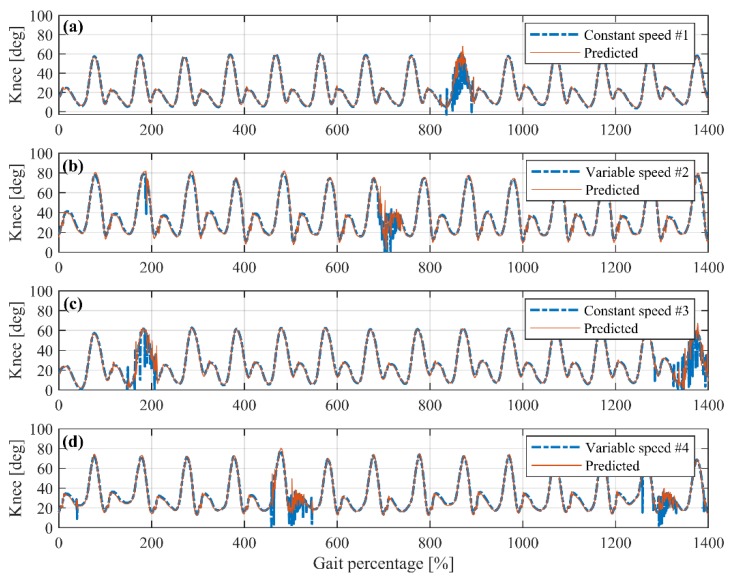
Reliability analysis of BP neural network continuous prediction.

To evaluate the prediction reliability of the proposed BP neural network, this paper studied the continuous gait prediction effects of several test subjects. In order to verify the anti-failure performance of the proposed method, the thigh IMU and the shank IMU in the data recording devices are randomly disabled within one or two cycles to simulate the malfunction of the prosthetic attitude measurement system. Figure 16 depicted some of the continuous test results.

Figure 16a shows the continuous prediction of a test subject waking at the constant speed. The IMU in the ninth cycle is randomly disabled to simulate incorrect knee angles caused by sensor malfunctions. As can be seen from the first eight cycles, the proposed method has a very good predictive effect in the constant-speed walking. The predicted knee angles almost coincide with the actual angle curve. In the ninth cycle, the input knee angles become invalid. The prediction of the proposed method will oscillate around the expected one, which means adding a filter in the subsequent process can simply and effectively handle this kind of short-term sensor malfunctions. In the tenth cycle, the input knee angle signal returns to normal, and the proposed method can quickly restore accurate continuous prediction for subsequent cycles. Figure 16c shows the gait prediction of another test subject at constant waking speeds. Two cycles are modified as malfunctioned. The prediction outcomes are also accurate and can recover from simulated sensor failures quickly.

Figure 16b shows the continuous prediction of a test subject walking at variable speeds. The second cycle is added with one sensor error, and the seventh cycle is simulated as sensor malfunctions. It can be obtained that the proposed method can handle accelerated and decelerated walking very well. The predicted peak/lowest stance/swing knee angle is very close to that in each cycle. Sensor sample errors and short-term malfunctions will not affect the prediction effect of this method. Figure 16d shows the prediction of the variable speed walking gait of another subject. More sensor errors and malfunctions are added for performance evaluation. It can be obtained from the result that the proposed BP neural network gait prediction method is effective and can handle both the constant-speed and variable-speed walking effectively and satisfactorily.

### 4.3. Experimental Layout

**Figure 17:**
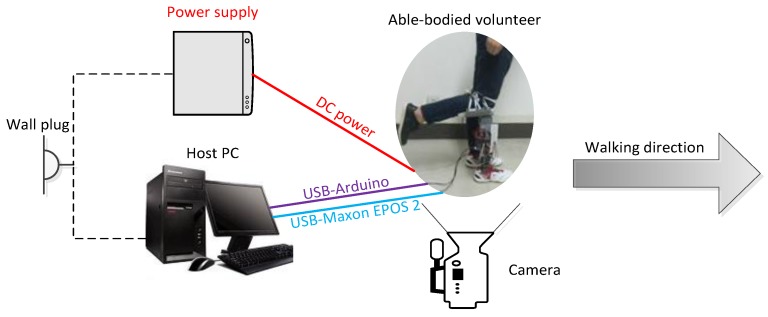
Experimental layout of the powered GFB prosthetic knee.

To experimentally verify the neural network control, an able-bodied subject (height: 178 cm, weight: 63 kg, age: 22, the written consent form was obtained prior to experiments) participated in the test of the GFB prosthetic knee via a particular connecting apparatus by flexing his knee to 90°, as shown in Figure 17. The GFB prosthetic knee is directly supplied with power through a dedicated DC power supply (DP712, Rigol, China). Experimental ankle trajectories and knee angles are collected by the camera (G920, Logitech, Switzerland) via video post-processing.

**Figure 18:**
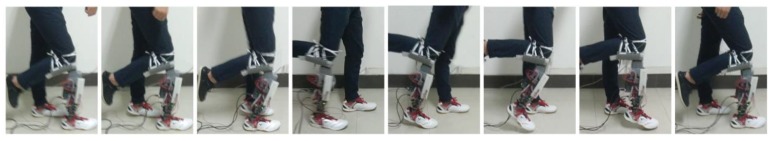
One gait cycle of the experimental level-ground walking.

After multiple times of training, the volunteer becomes familiar with wearing the GFB prosthetic knee to walk. Figure 18 shows one gait cycle of the experimental level-ground walking. To demonstrate the effectiveness of our proposed neural network control, specific self-selected speed and variable speed experiments are tested.

### 4.4. Constant-Speed Experiment

**Figure 19:**
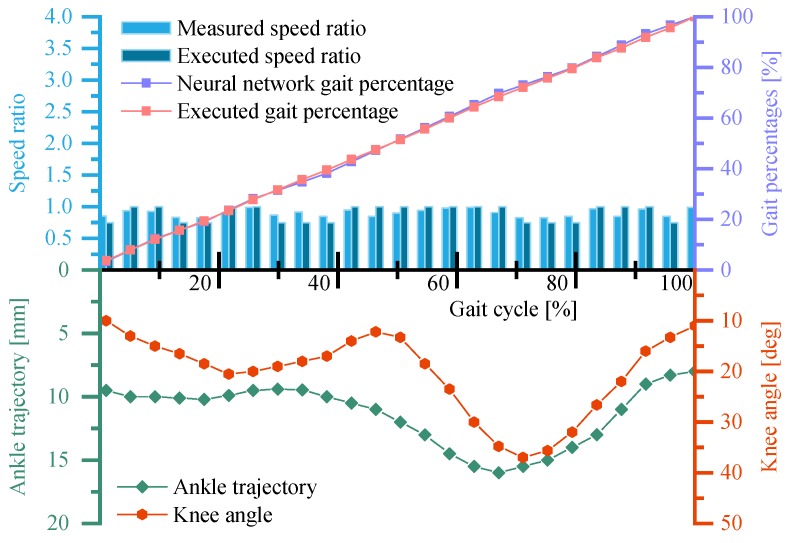
Running states of the neural network control system and the ankle trajectory/knee angle curve of the GFB prosthetic knee during the self-selected speed experiment.

The volunteer is asked to walk at his habitual speed (about 1.2 m/s). Figure 19 shows the running states of the neural network control system, the ankle trajectory, and knee angles of the volunteer in one steady gait cycle within continuous trials.

It can be obtained from Figure 19 that the measured speed ratio during the whole gait cycle is around or below K=1, which is consistent with the fact that the volunteer is walking at speed slightly slower than the reference speed of the GFB prosthetic knee (1.3 m/s). Due to the self-regulation of the neural network control system, the executed speed ratio of the GFB prosthetic knee varies between K=1 and K=0.75 to maintain the required walking speed. The executed gait percentage is close to the gait percentage predicted by the neural network, which verified the robustness of the overall control system. In addition, the ankle trajectory and the knee angle curve are close to the expected gait of the volunteer himself, which proves that the GFB prosthetic knee can provide proper level-ground walking gait.

### 4.5. Variable-Speed Experiment

**Figure 20:**
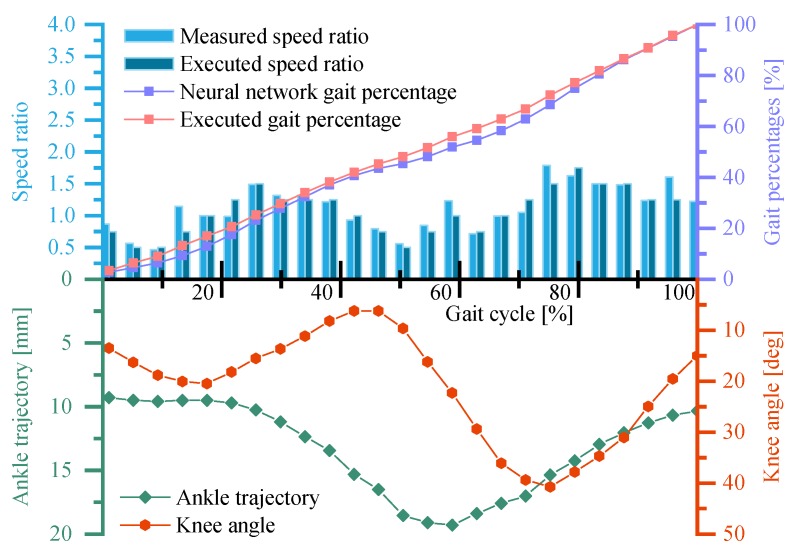
Running states of the neural network control system and the ankle trajectory/knee angle curve of the GFB prosthetic knee during the variable speed experiment.

To further demonstrate the effectiveness of the neural network control system, the volunteer is asked to vary his walking speed by acceleration and deceleration. Figure 20 shows the running states of the neural network control system as well as the ankle trajectory and the knee angles of the volunteer in one variable speed gait cycle within continuous trials.

It can be obtained from Figure 20 that the executed speed ratio of the GFB prosthetic knee fluctuates between K=0.5 and K=1.75 in the whole gait cycle and two peak speed ratios occur in the middle of both the stance phase and the swing phase. This indicates that the volunteer accelerated the prosthetic knee in the early stance/swing phase and then tried to decelerate the prosthetic knee in the late stance/swing phase. Because the pre-trained neural network can deal with accelerated/decelerated walking gait, the executed speed ratio generated by the neural network can follow the variable-speed walking well. The small deviation between the neural network predicted gait percentage and the executed gait percentage of the prosthetic knee is caused by the insufficient actuating torque of the cam-based nonlinear series elastic actuator when the speed ratio exceeds K=1. Compared with Figure 19, the amplitudes of the ankle trajectory and the knee angle curve of the volunteer are slightly larger, which is in line with the gait of variable speed walking.

## 5. Conclusions

This paper presented a speed-adaptive neural network control of a powered GFB prosthetic knee. The control system consists of the attitude measurement system and the neural network motor control system, which are run in parallel to reduce hardware/software requirements. To enable speed-adaptive asymmetrical walking gaits such as acceleration or deceleration, this paper only uses sensor data from the prosthetic knee, rather than data from both the sound and unsound legs. The current prosthetic knee gait can be recognized by the Gaussian mixture model-based gait classifier. Unlike conventional prediction method, the neural network in this paper does not output the predicted prosthetic knee motion, but the predicted gait percentage of the prosthetic knee. The specific prosthetic knee motion is obtained by matching the predicted gait percentage with the ideal gait database that stored gait data of different speeds. The benefit of doing this is that the size of the neural network can be reduced, and gait diversity can be increased and modified easily for customization. Level-ground walking experiments are conducted via the able-bodied subject. The results show that the neural network control system can successfully realize the speed-adaptive level-ground walking of the GFB prosthetic knee. Future work of this paper will extend the speed-adaptive control system suitable for ramp and stair walking, as well as the embedded transplantation into the development board.

## Figures and Tables

**Table 1:** Specifications of the powered geared five-bar (GFB) prosthetic knee.

Height	Weight (without Battery)	Max. Joint Torque	Max. Joint Speed	Max. Range of Motion
0.2 m	2.8 kg	77 N·m	1.85 rad/s	2.45 rad

**Table 2:** Data structures of the transmitted data from the attitude measurement platform.

Header	Yaw Float (4 Byte)	Pitch Float (4 Byte)	Roll Float (4 Byte)	Phase Flag (1 Byte)
0x69/0x0c	Y Byte 1,2,3,4	P Byte 1,2,3,4	R Byte 1,2,3,4	1/0

**Table 3:** Subjects information in collecting gait data.

No.	Gender	Weight	Height	Age	No.	Gender	Weight	Height	Age
1	male	89 kg	1.85 m	29	6	male	60 kg	1.80 m	25
2	male	70 kg	1.75 m	28	7	female	55 kg	1.64 m	33
3	female	60 kg	1.72 m	25	8	male	105 kg	1.76 m	61
4	female	48 kg	1.55 m	19	9	female	52 kg	1.72 m	41
5	male	53 kg	1.66 m	24	10	female	79 kg	1.62 m	32

**Table 4:** Statistical features of gait signals.

Name	Expression	Name	Expression
Mean	$\mu =\sum_{i=1}^{n}x_{i}/n$	Coefficient of Variation	σ/μ
Median	$x_{\frac{n+1}{2}}^{sort,\mathrm{odd}\;n},\left(x_{\frac{n}{2}}^{sort,\mathrm{even}\;n}+x_{\frac{n}{2}+1}^{sort,\mathrm{even}\;n}\right)/2$	Skewness	$\sum_{i=1}^{n}{\left(x_{i}-\mu \right)}^{3}/\left(n\sigma^{3}\right)$
Mode	$\underset{1\leq i\leq n}{\max}f\left(x_{i}\right),f=\mathrm{frequency}$	Kurtosis	$\sum_{i=1}^{n}{\left(x_{i}-\mu \right)}^{4}/\left(n\sigma^{4}\right)$
Total Distance	$\underset{1\leq i\leq n}{\max}x_{i}-\underset{1\leq i\leq n}{\min}x_{i}$	Slope Count	$\sum_{i=1}^{n-1}(x_{i+1}-x_{i})$
Variance	$\sum_{i=1}^{n}{\left(x_{i}-\mu \right)}^{2}/n$	Slope Zero Crossing	$C_{i=1}^{n-2}\left((x_{i+1}-x_{i})\left((x_{i+2}-x_{i+1}\right)\leq 0\right),C=\mathrm{count}$
Standard Deviation	$\sigma =\sqrt{\sum_{i=1}^{n}{\left(x_{i}-\mu \right)}^{2}/\left(n-1\right)}$		
